# Sibling Violence and Bullying Behaviors in Peers: The Mediational Role of Self-Esteem

**DOI:** 10.3390/ijerph21020227

**Published:** 2024-02-15

**Authors:** Catarina Pinheiro Mota, Joana Rita Sousa, Inês Carvalho Relva

**Affiliations:** 1 Department of Education and Psychology, University of Trás-os-Montes and Alto Douro (UTAD), Quinta de Prados, 5000-801 Vila Real, Portugal; joana_rita_04@hotmail.com (J.R.S.); irelva@utad.pt (I.C.R.); 2 Center for Psychology at the University of Porto (CPUP), R. Alfredo Allen, 4200-135 Porto, Portugal; 3 Research Center in Sports Sciences, Health Sciences and Human Development (CIDESD), Centre for Research and Intervention in Education (CIIE), University of Porto, 4200-135 Porto, Portugal

**Keywords:** sibling violence, bullying, self-esteem, victims, aggressors

## Abstract

In the context of the existing research on families, sibling violence is a less explored area. However, it has seemingly received more attention recently, and it can assume a relevant role in understanding the maladaptive behavior of youngsters and bullying. Additionally, adolescents involved in bullying and self-esteem are associated with disruptive violence inside the family context. This study’s sample consisted of 286 students, aged between 12 and 17 years, from both sexes. This study intends to explore the association between sibling violence and bullying behavior in peers and the mediator effect of self-esteem. The measures for data collection were a demographic questionnaire, the Social Exclusion and School Violence Questionnaire, The Revised Conflict Tactics Scales (Portuguese version for siblings), and the Rosenberg Self-Esteem Scale. The results show a negative effect between negotiation in the sibling relationship (victimization) and social exclusion and verbal aggression related to bullying behavior. Self-esteem represents a total and negative mediator in this connection. Our results also show a variety of indirect outcomes amongst the negotiation dimension, psychological aggression and injury between siblings, and the social exclusion and verbal aggression dimensions (on the aggression and victimization scales). The results will be discussed according to the attachment theory but considering the importance of affective bonds with siblings as a predisposing factor to an adaptive development course.

## 1. Introduction

According to Bowlby (1969) [1], the child is predisposed to build and develop emotionally significant close relationships with an adult, compromising the quality of the other relationships established in the life cycle. The availability and accessibility of the primary figures of attachment (usually the mother) to the needs of the child promotes the establishment of the internalization of this figure as a safe port and base, allowing the child to explore the outside world and imbuing them with the feeling of being safe [2]. The presence of appropriate care from the primary attachment figures transmits a sense of security to a child, stimulating the building of positive internal working models of oneself, others, and the world [1,3,4], which, in the sequence, is an important base for self-esteem development [2]. Establishing secure attachment relationships with parents has effects, although not permanently definitive ones, on all social relations the child establishes later [1,5]. These effects extend to all significant peer relationships, including those with siblings, although there can be differences in this respect. If there are secure bonds with parents, relationships between siblings and peers tend to be positive, unlike insecure bonds, which foster relationships based on aggressiveness or conflict [6,7].

Thus, the transmission of feelings of security may allow, in adolescence, the transmission of attachment functions [8,9,10,11,12] and the extension of relational networks (e.g., Rocha & Matos, 2012) [13]. In one’s close social environment, a sibling may initially act as a haven, someone to ask for advice and support in the anxious situations that characterize this developmental phase [1,5,14]. Peer relationships differ qualitatively from those with parents in that the first is parallel and the latter is complementary. In the sibling relationship dynamic, each sibling is simultaneously the caregiver and receiver of care, depending on the specific situation [15]; however, this reciprocity does not always occur. Sibling violence, even though it is considered a harmless form of familial aggression [16], tends to be more prevalent in the family context [17] when compared to other forms of violence.

Olweus (1993) [18] defines bullying as a set of negative actions carried out in a school environment and perpetrated by one or more students on another. These actions reach the status of bullying if they present a systematic character and intentionally aim to hurt or embarrass the other [14,19]. The imbalance of power is one of the characteristics of bullying since the aggressor tends to consider themselves superior to the victim, whether physically or psychologically [20,21]. For Olweus (1993) [18], acts of bullying are usually exercised only on one victim; however, they can also focus on a group of students.

Bullying can take several forms: (a) verbal bullying, reflected through insults and the use of derogatory nicknames; (b) physical and material bullying, exercised through hitting, pushing, stealing, destroying personal objects, or throwing objects at the victim; (c) psychological and moral bullying, perpetrated through humiliation, isolation, contempt, blackmail, and/or defamation; (d) sexual bullying, concretized by rape, sexual abuse, and/or harassment; and (e) virtual bullying, also referred to as cyberbullying, which is carried out with the use of digital technologies (mobile phone and the internet) [22]. Violence in bullying may be direct or indirect. Direct bullying is understood as all aggressions perpetrated with the effective presence of the victim, such as the verbal, physical, or material forms of bullying. Indirect bullying focuses on the social isolation of the victim (e.g., psychological and verbal), and the victim is not directly targeted in aggressive acts [18].

According to Sousa et al. (2011) [23], there are still distinct roles in the actors of bullying, highlighting the victim (who suffers from the aggression), victim/aggressor (alternate role of victim and aggressor), the aggressor (plays the role of the aggressor and only this role), and the observer (assists without participating either in the aggression or in the defense of the victim). According to Krienert and Walsh (2011) [24], there is consistent reciprocity in sibling violence, i.e., both play the alternating roles of victims and aggressors. The relationships in sibling violence contexts usually present power imbalances between the victim and aggressor (e.g., physical force) [25]. Differential parental treatment (e.g., sex differentiation in the education of children) [26], low parental supervision [27], and parental hostility [28] may be risk factors for sibling violence. The imbalance of power between victim and aggressor is more evident when the particularities of the performance of the role of each in the sibling dyad are not so distinct. The aggressors are represented as those who present characteristics of superiority (e.g., physical strength) toward the victims [29], and aggressors are usually male [30]. Males are also the ones who most perpetrate aggressions that cause serious physical harm [31]. According to Relva et al. (2014) [32], male siblings assaulting members of the same sex occurs more often than other types of assaults.

Regarding sibling violence victimization, psychological violence mainly affects females; however, in general, there are inconsistencies in the results regarding this topic [16,31,33]. For example, Dantchev et al. (2018) [34], in a sample of 6988 participants of the Avon Longitudinal Study of Parents and Children, a U.K. community-based birth cohort, females reported being victimized more often by a sibling than a male. For sibling bullying perpetration, no gender difference was found.

Caspi (2012) [35] identify various forms of aggression between siblings, namely at the physical, psychological, sexual, and even relational levels. Physical violence is violence that causes physical harm to the victim, while psychological violence tries to develop feelings of fear and ridicule [36]. Sexual violence, in turn, consists of sexual contact without the sibling’s consent [36]. However, the longer-term implications of this form of abuse are not entirely understood [37]. In relational violence, there is characteristically the proliferation of pejorative rumors about the victim [35] or exclusion [25], making this form of sibling abuse one that can have fatal consequences, such as siblicide (sibling homicide) [38].

Parke et al. (2001) [39] argue that conflict resolution tactics develop in the personal interrelationship between siblings and are later transferred to negotiation within one’s group of peer friends. The literature supports these conclusions, associating the conflicts that arise in the sibling context with those that occur in the peer context (e.g., [17,40,41,42,43,44]).

As previously mentioned, the literature points to a significant link between the presence of sibling violence and peer bullying (e.g., [37,40,42,45,46,47,48,49]). Tucker et al. (2014) [42] (p. 1604), in a study involving a sample of 1536 children aged between 3 and 9 years and 1523 adolescents aged between 10 and 17 years and data from the National Survey of Children’s Exposure to Violence (U.S.A.), found that “the number of children who exclusively experienced sibling victimization was greater in childhood (33%) compared to adolescence (14%) … and the proportion of individuals victimized by both siblings and peers was equivalent in childhood and adolescence (15%)”.

Also, the aggressors in sibling violence usually present a concomitant participation in bullying. Thus, in the school context, aggressor siblings tend to denounce acts of violence or occupy the role of victim/aggressor [17]. The same authors argue that the patterns apprehended in the family environment are later transferred to the school sphere. Thus, positive relationships in the peer group are expected regarding healthy relationships and the absence of sibling violence [50]. It should be noted that secure bonds with parents seem to contribute to the development of high levels of self-esteem (e.g., [51,52]). According to Serra (1988) [53], self-esteem consists of the judgment the subject makes of themselves and how they perceive the image that others and the world create of themselves. A positive judgment, in parallel with a perception that one can successfully perform the tasks one sets out to do consistently, results in high self-esteem. On the contrary, if the evaluation of the self is negative and accompanied by the perception that oneself is incapable of performing any task successfully, the indices of self-esteem are naturally low [53,54]. Also, Harter (1987) [55] proposed the concept of global self-esteem, which includes subgroups such as school-related and physical self-esteem. Regarding gender, according to Quatman and Watson (2001) [56], males have higher levels of overall self-esteem when compared with females; however, there are specificities of self-esteem that should be considered. Yeh and Lempers (2004) [50], in a study of 374 families from a Midwestern state in the U.S.A., suggest that siblings’ relationships influence the quality of their self-esteem. It should be noted that according to some studies [36,50], a common characteristic of sibling violence is low self-esteem. In addition, sibling violence seems to influence mental health, with well-being [28,57] and the low self-esteem of victims [14,58] or perpetrators [58] being mentioned in the literature. For example, in a retrospective study, Plamondon et al. (2021) [28] intended to explore whether sibling bullying victimization mediates the relationship between family dynamics during childhood and adolescence and young adults’ current well-being. The authors found that exposure to interparental conflict and sibling rivalry was associated with more experiences of sibling bullying and consequently associated with low levels of a sense of competence, self-esteem, satisfaction with life, and more internalized problems. More specifically, “any type of experience with sibling sexual abuse as a child negatively influences the self-esteem of college students” [59] (p. 209).

The results regarding the association between self-esteem and bullying behaviors could be more consistent. Álvarez-García et al. (2015) [60] conducted a systematic review to analyze the possible risk factors that predict the perpetration of bullying in adolescence. Their results suggest that self-esteem has an ambivalent relationship with bullying perpetration: “both low and high levels can predict an increased risk of bullying, and its interaction with other variables is the key to understanding its effect” [60] (p. 134). The way each adolescent experiences situations of violence may relate to their perception of themselves and others [61]. Adolescents with secure attachments to primary caregiver figures tend to have moderate to high levels of self-esteem, which facilitate the development of positive and secure relationships with their peer group [20,51,62,63].

On the other hand, adolescents involved in bullying situations, whatever role they assume, tend to have low levels of self-esteem [64,65,66]; more specifically, aggressors tend to have low self-esteem [21]. Aggressors even seem to resort to aggressive behavior to obtain a superior social status and popularity among peers [67,68]. Aggression offers some benefits regarding one’s perception of closeness and relationships with some peer groups [21].

Despite the importance of self-esteem in adolescents, to our knowledge, no studies from Portugal have explored the role of self-esteem between sibling violence and bullying in peers. It is still important to study them further in order to understand their association. To this extent, it is expected that the quality of the relationship between siblings and self-esteem can be associated with roles in bullying and positive relationships between siblings, and those absent of violence are associated with high levels of self-esteem, presenting themselves as a protective factor in the involvement of aggressive behaviors with peers in the school context.

### Objectives and Hypotheses

**Figure 1:**
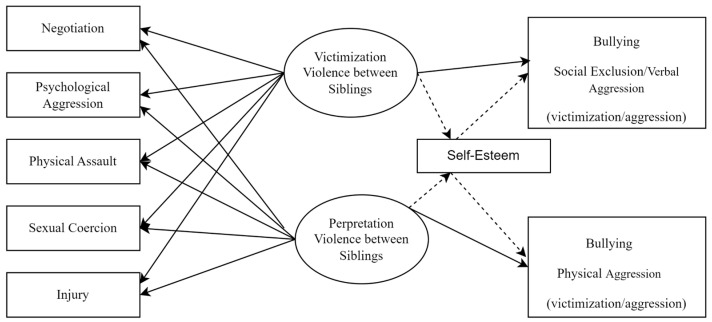
Conceptual Model.

This study aims to analyze the role of sibling violence (victimization and perpetration view) and self-esteem in bullying behaviors in adolescents, according to the victimization and aggression dimensions. It also intends to verify whether self-esteem mediates the association between sibling violence and bullying (see Figure 1). Considering the aims outlined above, we expected negotiation (sibling violence—victimization and perpetration) to have a negative association with social exclusion/verbal aggression and physical aggression (on victimization and aggression dimensions) and psychological aggression, physical assault, sexual coercion, and injury (sibling violence—victimization and perpetration), while we also expected it to have a positive association with social exclusion/verbal aggression and physical aggression (victimization and aggression dimensions). It was also expected that the mediating role of self-esteem has a reducing role in bullying behavior.

## 2. Materials and Methods

### 2.1. Participants

This study used data from a larger project that focuses on sibling violence, self-esteem, and bullying behaviors in adolescents while also analyzing sociodemographic variables. Detailed methodology descriptions can be found elsewhere [69]. In the present study, which is concerned with victimization, aggression, and bullying behavior, a sub-sample of 286 adolescents aged between 12 and 17 years participated (M = 13.55; SD = 1.12). Of these 286 adolescents, 115 (40.2%) were male, and 171 (59.8%) were female. Of the total respondents, 83 subjects (29%) attended the 7th grade, 89 (31.1%) attended the 8th grade, and 114 (39.9%) attended the 9th grade. In the present study’s sample, all the young people had siblings, ranging from 1 to 5 siblings (M = 2.1; SD = 0.76), totaling 174 male and 167 female siblings.

### 2.2. Measures

To conduct this study, a questionnaire was elaborated to assess the demographic characteristics revealed in the respondents’ characterization, considering the pre-defined objectives. Information was provided by the respondents (e.g., age, number, and position in the sibling dyad) and their parents (e.g., age, level of school, socioeconomic status).

To assess bullying, the Social Exclusion and School Violence Questionnaire was used (QEVE; [70], an adaptation of Martins, 2005; 2009; 2010) [71,72,73]. This is a self-report questionnaire subdivided into three subscales: Victimization, Aggression, and Observation of victimization/aggression. The victimization subscale consists of two factors: (1) social exclusion and verbal aggression and (2) physical aggression. The aggression subscale covers the same factors: (1) social exclusion and verbal aggression and (2) physical aggression. Finally, the observation of victimization/aggression subscale integrates three factors: violence with minor aggression, social exclusion and verbal aggression, and violence with severe aggression. In this questionnaire, answers are presented on a 4-point Likert scale (1 = never and 4 = almost always).

The internal consistency values evaluated through Cronbach’s alpha were acceptable (victimization = 0.85; aggression = 86). The same happened for the dimensions (social exclusion and verbal aggression in victimization = 0.85; physical aggression in victimization = 0.79; social exclusion and verbal aggression in aggression = 0.80, physical aggression in aggression = 0.96). Based on this study’s aims, the observation dimension was not used. Our confirmatory factor analysis indicated that the adjustment of the data to the proposed theoretical model led to the following values: SRMR = 0.06, CFI = 0.94, RMSEA = 0.08, χ^2^ (165) = 489.154, p < 0.001, χ^2^/df = 2.96, alpha = 0.91.

Our evaluation of sibling violence was carried out using The Revised Conflict Tactics Scales-Sibling Version (CTS2-SP; [31]), specifically a version adapted from the Conflict Tactics Scales 2 (CTS2-SP; [74]). This is a set of self-report scales directed toward subjects with one or more siblings, questioning them about the potential aggressive behaviors they have experienced within their sibling relationship. The scale is subdivided into two subscales: victimization and perpetration. Each subscale has five dimensions: negotiation, psychological aggression, physical assault, sexual coercion, and injury. The items appear paired, questioning the respondent on whether they fit within the role of the victim and/or the role of the aggressor. Answers are given on a seven-point Likert scale (Never happened/did not happen in the last year = 0; 1 = once in the previous year; 2 = twice in the last year; 3 = three to five times in the previous year; 4 = six to ten times in the last year; 5 = eleven to twenty times in the previous year; 6 = more than twenty times in the last year).

The analysis of internal consistency performed in this study presents acceptable values for both of the general scales (Cronbach’s alpha values of 0.78 for perpetration and 0.77 for victimization) and for the scales of perpetration and victimization (Cronbach’s alpha values of 0.79/0.84—negotiation, 0.87/0.79—psychological aggression, 0.84/0.88—physical assault, 0.67/0.58—sexual coercion, and 0.55/0.50—injury). Through our confirmatory factor analyses, we verified the adequacy of the data to the proposed theoretical structure with adequate adjustment indexes: perpetration—SRMR = 0.07, CFI = 0.92, RMSEA = 0.09, χ^2^ (75) = 246.65, *p* < 0.001, χ^2^/df = 3.29; victimization—SRMR = 0.06, CFI = 0.93, RMSEA = 0.08, χ^2^ (75) = 227.60, *p* < 0.001, χ^2^/df = 3.03.

The Rosenberg Self-Esteem Scale (R.S.E. [75]; adapted by [76]) was used to assess global self-esteem levels. This scale is a self-report instrument that aims to assess global self-esteem. This scale uses a 4-point Likert-type response format, with 1 meaning strongly disagree and 4 meaning strongly agree. Our internal consistency analysis for these data showed a Cronbach’s alpha of 0.84. Our confirmatory factor analysis presented adequate adjustment indices: SRMR = 0.05, CFI = 0.98, RMSEA = 0.06, χ^2^ (32) = 66.104, *p* < 0.001, χ^2^/df = 2.07.

### 2.3. Procedure

This study is part of a broader project that aims to analyze whether attachment to peers and violence between siblings and peers are associated with bullying behavior in young people while controlling for the role of self-esteem and the relationship between sibling dyads in terms of the roles of aggressor and victim. The procedures used in this study followed the General Regulation on Data Protection of the European Union and the Code of Ethics and Deontology for research of the Portuguese Psychologists Association. An authorization request was made to the Directorate General for Education (D.G.E.) through the Monitoring of Surveys in the School Environment platform to implement the study protocol in a school-based context. After receiving approval from the D.G.E., the sample was collected in the center and north of Portugal between January and March 2017, with students of the 7th, 8th, and 9th grades forming the sample. Requests for authorization to participate in the study were made to the schools in the referenced areas. Meetings were scheduled with the directors of the schools that agreed to participate to present the study protocol and alleviate any possible doubts they may have had. Informed consent was requested from the participants’ parents. The protocol was applied via the collaboration of the educational institutions, and the standard instructions for completing the investigation protocol were given to the students. Information was given to the students regarding confidentiality and voluntary participation in the study.

### 2.4. Statistical Analyses

This study is a cross-sectional study regarding its method of data collection. The sample size was tested using G*Power 3.1.9.7, considering the type of analyses envisaged in the study, with an effect size of d = 0.5, a significance level of 0.05, and a power of 0.95, along with five predictors, providing a minimum of 138 participants. The processing of data related to the construction of the database, exclusion of missing values and existing extreme values [77], and observation of the values of normality of distribution (skewness and kurtosis [78]) were carried out using the SPSS (Statistical Package for Social Sciences) version 28 (Windows). The scale scores, measured using Likert scales, were estimated through the averages of the responses (see Table 1). We conducted 1st order confirmatory factorial analyses (C.F.As) for the results derived from the use of the instruments. Correlation analyses (Pearson’s correlations), as preliminary analyses, were performed. Path analysis models tested the mediating effect of self-esteem in the association between sibling violence–perpetration/victimization and bullying among peers [79]. Several steps have been developed in the testing of mediation, namely the role of direct and indirect effects, considering the principles of the Sobel test. The path analysis models were ran through the AMOS (28 version) program. All results were analyzed based on a significance value of *p* < 0.05. Our C.F.As and the models were evaluated using the chi-square test, CFI, and RMSEA. The reference values for acceptable adjustment values were CFI ≥ 0.90 and RMSEA < 0.10 [79].

## 3. Results

### 3.1. Preliminary Analyses

Pearson’s correlations between self-esteem, sibling violence (perpetration and victimization), and bullying behavior (aggression and victimization) were calculated as part of our preliminary analyses.

The association between self-esteem and the perpetration of sibling violence was observed to be a positive and significant correlation of low magnitude for negotiation (*r* = 0.21, *p* < 0.001), and negative and significant correlations of low magnitude were found for psychological aggression (*r* = −0.19, *p* < 0.001), physical assault (*r* = −0.18, *p* < 0.001), sexual coercion (*r* = −0.15, *p <* 0.05), and injury (*r* = −0.18, *p* < 0.001).

On the association between self-esteem with victimization of sibling violence, there is a positive and significant correlation of low magnitude between negotiation (*r* = 0.24, *p* < 0.001), while psychological aggression (*r* = −0.19, *p* < 0.001), physical assault (*r* = −0.18, *p* < 0.001), and injury (*r* = −0.21, *p* < 0.001) all had negative and significant correlations of low magnitude. Sexual coercion is the only dimension that is not statistically significantly correlated.

Concerning bullying behavior (victimization), there is a negative and significant correlation between social exclusion and physical aggression with self-esteem (*r* = −0.21, *p* < 0.001; *r* = −0.14, *p* < 0.05), as well as a negative and significant correlation between physical aggression and negotiation (both perpetration and victimization) (*r* = −0.17, *p* < 0.001; *r* = −0.18, *p* < 0.001). Regarding bullying behavior (aggression), the results demonstrated a negative and significant correlation between social exclusion and self-esteem and negotiation (victimization) (*r* = −0.15, *p* < 0.05; *r* = −0.12, *p* < 0.05), as well as a positive and significant correlation between social exclusion and physical assault (both aggression and victimization) (*r* = 0.14, *p* < 0.05; *r* = 0.17, *p* < 0.001) and psychological aggression (both aggression and victimization) (*r* = −0.17, *p* < 0.001; *r* = −0.16, *p* < 0.001). Finally, we observed a positive significant correlation between physical aggression and sexual coercion (perpetration) (*r* = 0.37, *p* < 0.001). The results of the interscale correlations and the respective means and standard deviations are presented in Table 1.

### 3.2. Sibling Violence’s Impact on Bullying Behaviors: The Mediating Role of Self-Esteem

Path analysis models were used to analyze the effect of violence between siblings on bullying behaviors, with self-esteem being used as the mediating variable.

In the initial model referring to the subscale perpetration of sibling violence (Figure 1), we observed that the negotiation dimension negatively predicts the physical aggression dimension of the bullying victimization subscale (β = −0.19). Negotiation also negatively predicts the social exclusion and verbal aggression of the bullying aggression subscale (β = −0.14).

We also found that the psychological aggression dimension of the perpetration of violence between siblings positively predicts the social exclusion and verbal aggression of the bullying aggression subscale (β = 0.24).

Sexual coercion carried out by siblings positively predicts the social exclusion and verbal aggression of the aggression of bullying (β = 0.15) and the physical aggression of the bullying aggression subscale (β = 0.50).

Finally, the injury dimension of the perpetration subscale of sibling violence negatively predicts the physical aggression of the bullying aggression subscale (β = −0.17).

**Figure 2:**
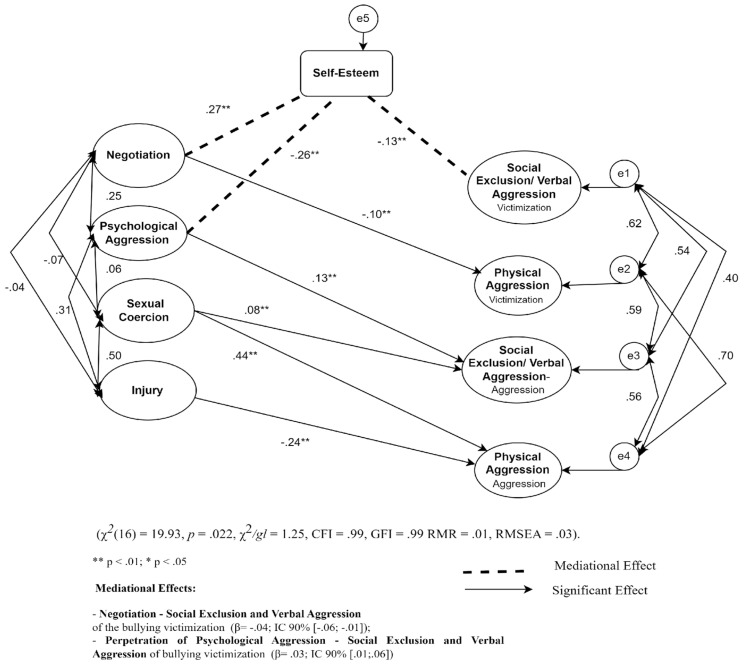
Representative model of the mediating role of self-esteem on the association between sibling violence—perpetration and bullying.

All previous correlations were maintained after introducing the self-esteem mediator variable, although their magnitude decreased (see Table 2). The existence of a mediating effect was verified, and the negotiation of violence between siblings was found to exert a negative indirect effect on the social exclusion and verbal aggression of bullying victimization through self-esteem (β = −0.04; I.C. 90% [−0.06; −0.01]); the perpetration of psychological aggression toward a sibling exerts a positive indirect effect on the social exclusion and verbal aggression of bullying victimization through self-esteem (β = 0.03; I.C. 90% [0.01; 0.06]) (Table 2) (Figure 2). As for the adjustment indices in the final model, these were as follows: χ^2^(16) = 19.93, *p* = 0.022, χ^2^/df= 1.25, CFI = 0.99, G.F.I. = 0.99 RMR = 0.01, RMSEA = 0.03 (Figure 2).

The initial model regarding victimization in sibling violence, the negotiation dimension (sibling violence victimization) negatively predicts physical aggression (victimization; β = −0.18). We also observed that negotiation negatively predicts social exclusion and verbal aggression from the bullying aggression subscale (β = −0.15).

However, after the introduction of the self-esteem mediator variable, there was a decrease in the significance of the initial direct effect of negotiation (sibling violence victimization) on social exclusion and verbal aggression of the bullying aggression subscale (βinitial = −0.15; βfinal = −0.04), thus verifying the total mediation of this variable (β = −0.02; I.C. 90% [−0.05, 0.00]).

It was also observed that the psychological aggression of the sibling violence victimization subscale exerts a positive indirect effect on the social exclusion and verbal aggression of bullying through the total mediation of self-esteem (β = 0.03; I.C. 90% [0.01, 0.05]).

**Figure 3:**
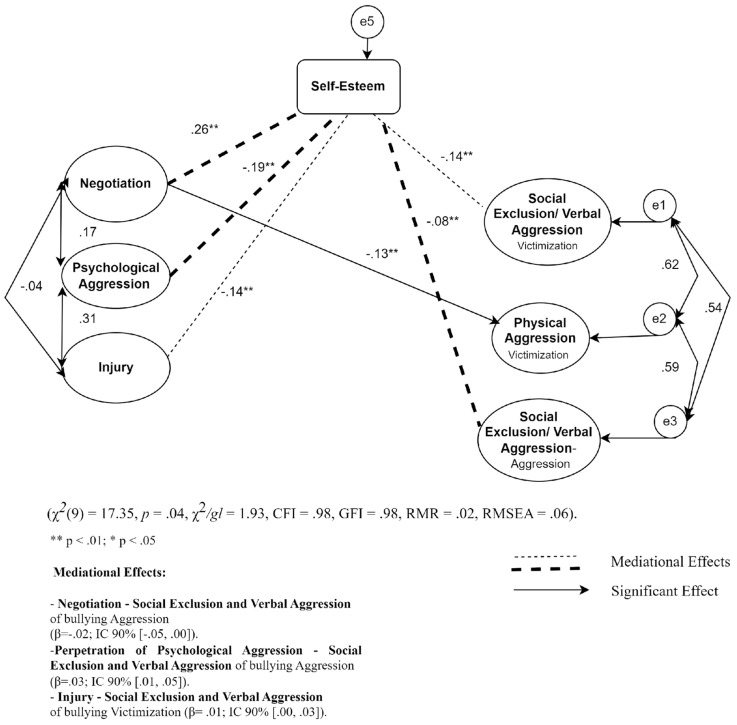
Representative model of the mediating role of self-esteem on the association between sibling violence—victimization and bullying.

Regarding the injury dimension of sibling violence victimization, it has a positive indirect effect on the social exclusion and verbal aggression of bullying victimization through the total mediation of self-esteem (β = 0.01; I.C. 90% [0.00, 0.03]) (Table 3) (Figure 3).

The final model suggests the following adjustment indices: χ^2^(9) = 17.35, *p* = 0.04, χ^2^/df = 1.93, CFI = 0.98, GFI = 0.98, RMR = 0.02, RMSEA = 0.06 (Figure 3).

## 4. Discussion

The main objective of the present study was to ascertain the effect of sibling violence on bullying behaviors (perpetration and victimization) and analyze the mediating role of self-esteem in the aforementioned associations.

In this sense, the results suggest that using negotiation as a strategy for resolving conflicts between siblings negatively predicts being a victim of physical aggression behaviors in bullying. Thus, negotiation to resolve conflicts between siblings seems to assume a protective role against involvement, as a victim, in physical aggressions perpetrated by peers. Competencies such as establishing oral communication, associated with debating skills, reinforce conflict resolution strategies using negotiation. As suggested by Brody (1998) [80] (p. 19), “psychosocial skills attained through sibling interactions are also used throughout life in a wide variety of other social relationships”, namely with peers.

The results suggest that the perpetration of psychological aggression (sibling violence) has a positive predictive effect regarding the aggressive behaviors of bullying, namely social exclusion and verbal aggression. Also, the perpetration of sexual coercion in sibling violence positively predicts bullying aggression, social exclusion, and verbal aggression and physical aggression. Regarding injury in the perpetration of violence between siblings, it was found to negatively predict acts of physical aggression in bullying. Regarding the perpetration of psychological and sexual aggression behaviors, these seem to exert an effect on the perpetration of aggressions centering around social exclusion and verbal aggression towards a particular victim in peers. In aggressors, involvement in sexual violence seems to potentiate physical violence behaviors. Thus, it can be argued that adopting the role of aggressor in a relationship between siblings (psychological and sexual aggressions) enhances the performance of the same role in bullying (social exclusion and verbal aggression and physical aggression). The literature also points to a relationship between sibling aggression and school bullying (e.g., [42,44,45,48,52]).

Additionally and as suggested by Updegraff et al. (2005) [27], the propensity of siblings to hurt in other interpersonal relationships may be associated with reduced levels of intimacy and involvement and negative relationships between siblings. These results were expected, to the extent whereby the divergences arising from social interaction between siblings are also adopted in resolving problems between peers [39]. Some authors have found similar results, namely when verifying whether aggressors in the context of sibling relationships also play the role of aggressors in the context of bullying situations between peers (e.g., [17,40,81]) and the positive association between sibling aggression victimization and bullying victimization [82]. Tippett and Wolke (2015) [17] also showed results comparable to ours in a study involving a sample of 4237 adolescents, verifying that aggressor siblings tend to occupy the role of victim/aggressor in bullying and that, otherwise, these siblings perpetrate and are victims of violence in their peer group.

It was verified, as expected, that self-esteem is a negative predictor of victimization behaviors related to social exclusion and verbal aggression in bullying. Thus, subjects with high self-esteem seem to be protected from becoming a victim of indirect aggressions. These data are corroborated by some other studies [64,65,83], the results of which indicate that there is a significant negative association between both variables, self-esteem, and peer victimization. In this sense, adolescents with a positive personal evaluation tend to carry out the proposed tasks, showing no need for involvement in conflict with peers (e.g., [67,68]). Oliva and Arranz (2005) [84] (p. 265) showed that, especially for girls, “a good relationship with their siblings was linked to good relationships with their parents and peers, as well as increased self-esteem and life satisfaction”.

Although there was no predictive effect of the use of negotiation and psychological aggression as a conflict resolution tactic in victimization, social exclusion, and verbal aggression, the results of the present study point to the existence of a total negative mediation of self-esteem in these associations. In this sense, negotiation, defined as a strategy of solution orientation, occurs when conflicts are resolved in mutually satisfying ways that reflect concern for the relationship [85], denoting the concern for oneself and the other, contributing to high self-esteem. This result has already been corroborated in the same sample in a more extensive study, so self-esteem seems to promote an increase in negotiation strategies in sibling violence, whether from the point of view of the victim or even the perpetrator [69]. Conversely, social problems with siblings have been linked to difficulties in emotional regulation [86], which can lead to low self-esteem. However, we believe that self-esteem is an important personal resource for psychosocial adjustment. When adolescents can build a positive image of themselves, there is no need to engage in aggressive behaviors such as, in this case, peer bullying, since, for an adolescent, peer aggression constitutes a form of self-affirmation, a conquest of power, and social status [67,68]. Dantchev and Wolke (2019) [19] suggest that high self-esteem is protective against becoming a sibling victim. Povedano et al. (2011) [87] point out that adolescents who display social and personal competencies towards their peers seem to protect themselves from the hostile behavior of aggressors who target vulnerable boys and girls as targets of violence. Studies suggest that high self-esteem is associated with lower bullying behaviors among siblings [50] and between peers [64,65]. In fact, in an earlier study with the same data, it was found that self-esteem was positively associated with the use of negotiation to solve sibling conflicts and negatively associated with psychological aggression, physical assault, and injury concerning both the perpetration and victimization of sibling violence [69]. In the same study, it was also verified that females showed a greater predisposition to establish conversations with their siblings when compared with males.

Regarding the model for victimization, the results indicate that the use of negotiation by siblings is a negative predictor in the dimensions of physical aggression (victimization). Thus, adolescents whose siblings adopt positive resolution strategies such as negotiation establish quality relationships with their siblings, which seems to have a protective effect on the occurrence of physical assault victimization. For example, a study by Truong et al. (2023) [57] found that children with positive sibling relationships were less bullied by their peers. An adolescent’s capacity to construct and maintain dialogues with their siblings fosters the ability to resolve conflicts through negotiation with peers. If, in the family dynamic, the sibling prefers dialogue in conflict resolution, they transfer this strategy from the family to the school environment [39]. To this extent, the quality of communication between siblings can give adolescents a greater capacity to manage their relationships with peers [88]. The literature corroborates the results presented, showing that lower levels of involvement in sibling violence among adolescents may be an indicator of greater emotional regulation, which is reflected in lower participation in peer bullying (e.g., [42,44,81]).

In the present study, self-esteem negatively predicted, once again, bullying behaviors among peers, namely victimization and aggression regarding social exclusion and verbal aggression. Thus, young people with high self-esteem tend to engage less in bullying behaviors, either victimization or aggression, through social exclusion and verbal aggression. A recent systematic review conducted by Agustiningsih et al. (2023) [89] (p. 1). suggests that “adolescent bullying and cyberbullying perpetration may be mitigated by higher levels of self-esteem”. High self-esteem promotes the widening of one’s social network [3], which may be relevant in establishing more adjusted social relations [13]. Therefore, less involvement in behaviors of social exclusion and verbal aggression, both as a victim and as an aggressor, seems to be associated with the establishment of positive relationships in one’s peer group.

Also, the total mediating effect of the self-esteem variable was observed for the victimization subscale of sibling violence. Thus, being a victim of both psychological aggression and injury (physical assaults causing severe physical harm) can have an effect on the victimization of social exclusion and verbal aggression through self-esteem. Previous studies carried out with a similar sample and involving a broader analysis have already discussed the role of self-esteem and its relationship with violence between siblings from the perspective of both the victim and the perpetrator. Self-esteem is a protector against psychological aggression, physical assault, and injury, both for the victim and in the perpetrator [69]. As mentioned earlier, the results of a study conducted by Tucker et al. (2019) [90] suggest that being a sibling victim leaves children and adolescents vulnerable to peer victimization. Thus, victimization due to psychological and physical aggression with severe damage seems to predict lower levels of self-esteem, which, in turn, predicts greater school victimization through social exclusion and verbal aggression. Thus, it is suggested that being a victim of aggressive behavior on the part of siblings predisposes the adolescent to low self-esteem [14,58]. As a result, the adolescent, considering themselves to be undeserving of love and affection, also becomes vulnerable to aggression by the peer group, being vulnerable to bullying [62,67]. As stated earlier, both victims [83,91,92] and bullies tend to have low self-esteem [91].

Finally, the total negative mediation of self-esteem was verified in the association between using negotiation (victimization) as a problem-solving strategy and aggression related to social exclusion and verbal aggression in bullying. These results are consistent with those found in previous studies, so negotiation, as a problem-solving strategy, solves problems in peer relationships [93]. This “cooperative solution of sibling conflict and a general sense of goodwill between siblings certainly can enhance children’s adjustment” [58] (p. 10), namely high self-esteem, which can be extended to peer relationships. In addition, siblings can learn from siblings to solve problems or negotiate, and this can contribute as a protective factor in other contexts [94]. Thus, it is suggested that healthy social interaction between siblings, namely the use of negotiation in conflict management, when associated with high levels of self-esteem, protects the adolescent against the perpetration of aggressive behaviors in the school environment [17]. These results align with those verified in [95], where it was stated that the social support received from siblings may serve as a protective factor in peer bullying. Also, Arslan (2016) [96] conducted a study to explore the mediating role of resilience and self-esteem in the associations between psychological maltreatment–emotional problems and psychological maltreatment–behavioral problems in adolescents. This author found that both variables appear to play a protective role in emotional problems and behavioral problems in psychologically maltreated individuals. It can also be hypothesized that age can contribute to this discussion. Negotiation strategies may be more prevalent due to age, which was not controlled for in this study, as older siblings exert more control in conflict resolution [85]. Age and dyad position influence sibling conflict resolution. Mota et al. (2023) [69] found significant differences in physical assault regarding the position in the sibling dyad. The middle siblings, as victims, were more often involved in acts of physical assault when compared with other positions (youngest or oldest). In addition, the choice to use negotiation to resolve conflicts that arise in sibling relationships allows the adolescent to develop positive feelings about themselves and others. In this sense, in the victimization model, it was verified that the quality of the relationship between siblings, guided by adaptive communication strategies (negotiation), predicts, through the positive image that young people build of themselves (self-esteem), the absence of maladjusted behaviors with peers (bullying).

## 5. Limitations, Practical Implications, and Suggestions for Future Studies

No study is without limitations. Thus, the primary limitation of this study is that its sample was taken at a single point in time; thus, this did not allow for the monitoring of the sample or the establishment of causal relationships. In addition, the protocol extension may have affected the coherence of the responses to the factor of tiredness. Also, relying only on self-reporting for data collection may constitute a limitation, and it is relevant to account for the perceptions of siblings and parents. The sample was partially recruited by convenience in a limited area from a territorial point of view, and it is important to extend it to the whole country. Some participants presented a reluctance to answer the questionnaire, specifically when faced with questions regarding the problem of sibling violence and bullying behavior, since it is a theme that is still not well accepted. Also, the small age range of our sample is important to note, as it hindered analyses including subjects of different ages. Also, in the main models, sex differences were not controlled for.

Finally, it is essential to highlight the practical issues implicit in this study. Thus, the importance of self-esteem in the adjusted psychosocial development of adolescents is of a high level, protecting them from situations of a violent nature, particularly regarding violence between siblings, as well as peer bullying in the school context. The predictive effect of sibling violence on bullying also deserves special attention. Despite it being difficult to diagnose aggression in siblings, due to its acceptance as a normal behavior, we do not mean to imply that it is less valued. The transfer of certain aggressive behaviors to the peer group deserves special attention because of the consequences they have for all involved. Given these implications, one should be alert to the importance of high levels of self-esteem, and awareness regarding the problems of sibling violence and peer bullying should be raised. In this sense, we suggest that it is essential to address both family and school contexts via psychosocial interventions for adolescents, and there is an interconnection between the family and school contexts.

In conclusion, in future studies, broadening the sample’s age range would be useful, as this would allow for a more detailed analysis of sibling violence and peer bullying. It would be interesting to carry out multi-group analyses of sex, as it is a relevant variable in the context of violence, both between siblings and in bullying behavior. Also, the extension of the study to other forms of intrafamily violence, as well as longitudinal studies, seems to be pertinent to note, as this would enable the establishment of cause–effect relationships between the various themes. In addition, the associated developmental differences could be analyzed. Given that only a single perspective was collected (that of the participants), to complement the information collected for this study, future studies could involve collecting data from siblings and parents regarding other elements using, for example, a qualitative methodology.

## 6. Conclusions

This study is relevant because it adds information to the existing literature, as sibling violence is not a topic that has been extensively discussed in scientific research. This work highlights not only the contextualization of the self-esteem variable, which has been associated with sibling violence, but more than that, its relationship between sibling violence and bullying. Few studies have linked sibling violence (from the perspective of the victim or perpetrator) and bullying behavior among peers (also from the perspective of the victim or aggressor). This study explores, on the one hand, the positive perspective of negotiating the violence between siblings and its relationship with reduced bullying behavior (physical aggression) and, on the other hand, the negative experience of violence between siblings (such as psychological aggression, sexual coercion, and injury) as promoters of bullying behavior (social exclusion/verbal aggression and physical aggression). As we expected, self-esteem acts as a protector against adversity in the lives of young people. Self-esteem has thus been shown in this study to protect young people who experience sibling violence from bullying behaviors, mainly social exclusion and verbal aggression. We believe that further studies could be carried out and that delving deeper into this subject could be relevant to preventing future maladaptive behavior.

## Figures and Tables

**Table 1:** *Correlation* between variables, mean, and standard deviation.

Variables (N = 286)	1	2	3	4	5	6	7	8	9	10	11	12	13	14	15
1. SELF-ESTEEM	-														
*SIBLING VIOLENCE* *Perpetration*															
2. Negotiation Sig. (2 tailed)	0.21 ** 0.000	-													
3. Psychological aggression Sig. (2 tailed)	−0.19 ** 0.001	0.25 ** 0.000	-												
4. Physical assault Sig. (2 tailed)	−0.18 ** 0.002	0.13 ** 0.034	0.74 ** 0.000	-											
5. Sexual coercion Sig. (2 tailed)	−0.15 * 0.014	−0.07 0.238	0.06 0.281	0.33 ** 0.000	-										
6. Injury Sig (2 tailed)	−0.18 ** 0.002	−0.04 0.482	0.31 ** 0.000	0.62 ** 0.000	0.51 ** 0.000	-									
*SIBLING VIOLENCE* *Victimization*															
7. Negotiation Sig (2 tailed)	0.24 ** 0.000	0.94 ** 0.000	0.16 ** 0.005	0.06 0.283	−0.07 0.247	−0.05 0.377	-								
8. Psychological aggression Sig (2 tailed)	−0.19 ** 0.002	0.25 ** 0.000	0.95 ** 0.000	0.73 ** 0.000	0.06 0.332	0.32 ** 0.000	0.18 ** 0.003	-							
9. Physical assault Sig (2 tailed)	−0.18 ** 0.003	0.13 * 0.023	0.73 ** 0.000	0.92 ** 0.000	0.30 ** 0.000	0.59 ** 0.000	0.07 0.276	0.73 ** 0.000	-						
10. Sexual coercion Sig (2 tailed)	−0.02 0.755	0.03 0.629	0.16 ** 0.007	0.43 ** 0.000	0.60 ** 0.000	0.59 ** 0.000	0.03 0.585	0.14 * 0.018	0.39 ** 0.000	-					
11. Injury Sig (2 tailed)	−0.21 ** 0.000	−0.01 0.863	0.33 ** 0.000	0.62 ** 0.000	0.51 ** 0.000	0.87 ** 0.000	−0.04 0.534	0.31 ** 0.000	0.64 ** 0.000	0.53 ** 0.000	-				
*BULLYING Victimization*															
12. Social Exclusion/ Verbal Aggression Sig (2 tailed)	−0.21 ** 0.000	−0.05 0.135	0.06 0.230	0.01 0.140	0.08 0.220	0.02 0.146	−0.06 0.260	0.04 0.150	0.02 0.167	−0.03 0.289	0.02 0.137	-			
13. Physical Aggression Sig (2 tailed)	−0.14 * 0.002	−0.17 ** 0.000	0.06 0.145	0.04 0.156	0.10 0.245	0.06 0.240	−0.18 ** 0.000	0.04 0.156	0.08 0.267	−0.02 0.350	0.06 0.210	0.62 ** 0.000	-		
*BULLYING Aggression*															
14. Social Exclusion/ Verbal Aggression Sig (2 tailed)	−0.15 * 0.001	−0.10 0.134	0.17 ** 0.001	0.14 * 0.002	0.15 * 0.001	0.11 0.156	−0.12 * 0.002	0.16 ** 0.000	0.17 ** 0.000	0.06 0.256	0.10 0.189	0.55 ** 0.000	0.60 ** 0.000	-	
15. Physical Aggression Sig (2 tailed)	−0.10 0.136	−0.10 0.145	−0.04 0.216	−0.04 0.217	0.37 ** 0.000	0.02 0.245	−0.10 0.267	−0.04 0.214	−0.02 0.277	−0.02 0.178	0.01 0.290	0.40 ** 0.000	0.67 ** 0.000	0.54 ** 0.000	-
*M*	3.01	3.04	1.09	0.38	0.08	0.17	2.91	1.02	0.40	0.07	0.18	1.27	1.10	1.22	1.04
*SD*	0.59	1.68	1.20	0.65	0.30	0.40	1.66	1.19	0.69	0.27	0.43	0.41	0.22	0.29	0.23

Note: * *p* < 0.05; ** *p* < 0.01.

**Table 2:** Model coefficients regarding sibling violence (perpetration) in bullying behavior: the mediational role of self-esteem.

Sibling Violence Perpetration			Estimate	S.E.	C.R.	*p*-Value
DIRECT EFFECTS						
Negotiation	→	Self-Esteem	0.270	0.056	3.342	***
Psychological Aggression	→	Self-Esteem	−0.260	0.071	3.967	***
Self-Esteem	→	Social Exclusion/Verbal Aggression (victimization)	−0.131	0.073	2.818	***
Negotiation	→	Physical Aggression (victimization)	−0.100	0.040	2.076	***
Psychological Aggression	→	Social Exclusion/Verbal Aggression (aggression)	0.132	0.072	1.510	***
Sexual Coercion	→	Social Exclusion/Verbal Aggression (aggression)	0.083	0.015	1.123	0.003
Sexual Coercion	→	Physical Aggression (aggression)	0.441	0.083	4.556	***
Injury	→	Physical Aggression (aggression)	−0.242	0.045	3.599	***
MEDIATIONAL MODEL						
Negotiation	→	Self-Esteem	0.270	0.056	3.342	***
Psychological Aggression	→	Self-Esteem	−0.260	0.071	3.967	***
Self-Esteem	→	Social Exclusion/Verbal Aggression (victimization)	−0.131	0.073	2.818	***
INDIRECT EFFECTS						
Negotiation	→	Social Exclusion/Verbal Aggression (victimization)	−0.040			***
Psychological aggression	→	Social Exclusion/Verbal Aggression (victimization)	0.030			***

Note. *** *p* < 0.001.

**Table 3:** Model coefficients of sibling violence (victimization) in bullying behavior: the mediational role of self-esteem.

Sibling Violence Victimization			Estimate	S.E.	C.R.	*p*-Value
DIRECT EFFECTS						
Negotiation	→	Self-Esteem	0.260	0.046	3.152	***
Psychological Aggression	→	Self-Esteem	−0.190	0.053	3.651	***
Self-Esteem	→	Social Exclusion/Verbal Aggression (victimization)	−0.142	0.062	2.704	***
Negotiation	→	Physical Aggression (victimization)	−0.130	0.066	2.357	***
Self-Esteem	→	Social Exclusion/Verbal Aggression (aggression)	−0.081	0.032	1.110	0.003
Injury	→	Self-Esteem	−0.142	0.035	1.895	***
MEDIATIONAL MODEL						
Negotiation	→	Self-Esteem	0.260	0.046	3.152	***
Psychological Aggression	→	Self-Esteem	−0.190	0.053	3.651	***
Injury	→	Self-Esteem	−0.142	0.035	1.895	***
Self-Esteem	→	Social Exclusion/Verbal Aggression (victimization)	−0.142	0.062	2.704	***
Self-Esteem	→	Social Exclusion/Verbal Aggression (aggression)	−0.081	0.032	1.110	0.003
INDIRECT EFFECTS						
Negotiation	→	Social Exclusion/Verbal Aggression (aggression)	−0.022			***
Psychological Aggression	→	Social Exclusion/Verbal Aggression (aggression)	0.030			***
Injury	→	Social Exclusion/Verbal Aggression (victimization)	0.012			***

Note. *** *p* < 0.001.

## Data Availability

The data are not publicly available due to privacy reasons.
